# Dimethylsulfoniopropionate Sulfur and Methyl Carbon Assimilation in *Ruegeria* Species

**DOI:** 10.1128/mBio.00329-20

**Published:** 2020-03-24

**Authors:** Joseph S. Wirth, Tao Wang, Qiuyuan Huang, Robert H. White, William B. Whitman

**Affiliations:** a Department of Microbiology, University of Georgia, Athens, Georgia, USA; b Department of Biochemistry, Virginia Polytechnical Institute and State University, Blacksburg, Virginia, USA; University of Southern California

**Keywords:** *Ruegeria*, cysteine biosynthesis, dimethylsulfoniopropionate, isotope labeling, methanethiol, methionine

## Abstract

Dimethylsulfoniopropionate (DMSP) is abundant in marine environments and an important source of reduced carbon and sulfur for marine bacteria. While both Ruegeria pomeroyi and Ruegeria lacuscaerulensis possessed genes encoding the DMSP demethylation and cleavage pathways, their responses to DMSP differed. A glucose-fed, chemostat culture of *R. pomeroyi* consumed 99% of the DMSP even when fed a high concentration of 5 mM. At the same time, cultures released 19% and 7.1% of the DMSP as dimethylsulfide (DMS) and methanethiol, respectively. Under the same conditions, *R. lacuscaerulensis* consumed only 28% of the DMSP and formed one-third of the amount of gases. To examine the pathways of sulfur and methyl C assimilation, glucose-fed chemostats of both species were fed 100 μM mixtures of unlabeled and doubly labeled [*dimethyl*-^13^C, ^34^S]DMSP. Both species derived nearly all of their sulfur from DMSP despite high sulfate availability. In addition, only 33% and 50% of the methionine was biosynthesized from the direct capture of methanethiol in *R. pomeroyi* and *R. lacuscaerulensis*, respectively. The remaining methionine was biosynthesized by the random assembly of free sulfide and methyl-tetrahydrofolate derived from DMSP. Thus, although the two species possessed similar genes encoding DMSP metabolism, their growth responses were very different.

## Abstract (precis)

Dimethylsulfoniopropionate (DMSP) is abundant in marine environments and an important source of reduced carbon and sulfur for marine bacteria. DMSP is the precursor for the majority of atmospheric dimethylsulfide (DMS), a climatically active gas that connects the marine and terrestrial sulfur cycles. Although research into the assimilation of DMSP has been conducted for over 20 years, the fate of DMSP in microbial biomass is not well understood. In particular, the biosynthesis of methionine from DMSP has been a focal point, and it has been widely believed that most methionine was synthesized via the direct capture of methanethiol. Using an isotopic labeling strategy, we have demonstrated that the direct capture of methanethiol is not the primary pathway used for methionine biosynthesis in two *Ruegeria* species, a genus comprised primarily of globally abundant marine bacteria. Furthermore, although the catabolism of DMSP by these species varied greatly, the anabolic pathways were highly conserved.

## INTRODUCTION

Dimethylsulfoniopropionate (DMSP) is abundant in marine surface waters. In the North Sea, the concentration of DMSP cycles seasonally from micromolar levels in the summer to picomolar levels in the spring and fall (1, 2). The majority of marine DMSP comes from halophytic plants and algae, where it is believed to regulate osmotic pressure but may also provide antioxidant, predator deterrent, and/or cryoprotectant functions (2). There is also evidence that at least 0.5% of marine bacteria are capable of producing DMSP (3). Consistent with its role in osmoregulation, plants that produce the most DMSP are generally halotolerant and of marine origin, with sugarcane being the only nonmarine exception (4, 5). During ^35^S-labeling studies with the protist Oxyrrhis marina, approximately 15% of added DMSP accumulated intracellularly but was not metabolized (6). Molar levels of intracellular DMSP have been observed in some organisms, and it is estimated that up to 10% of the total fixed carbon in the ocean is in the form of DMSP (5). Furthermore, DMSP released from phytoplankton blooms can satisfy up to 3% to 15% of the microbial carbon demand and 30% to 100% of the microbial sulfur demand (5, 7, 8).

DMSP is the precursor for the majority of atmospheric dimethylsulfide (DMS), which is a climatically active gas and connects the marine and terrestrial sulfur cycles (8, 9). It was previously believed that H_2_S was responsible for the transfer of sulfur between marine and terrestrial environments, but the necessary atmospheric concentrations were never detected, and the surface layers of the ocean are too oxidizing to sustain an equilibrium with the atmosphere (9). However, the concentration of DMS in marine surface layers is sufficiently high, and DMS is resistant to oxidation in the lower atmosphere (9). Its oxidation in the troposphere by radicals formed by photolysis such as OH˙ and NO_3_˙ produces sulfur species that can be transferred to terrestrial environments via rain and promote the formation of cloud condensation nuclei, resulting in an increased albedo effect and global cooling (2, 5, 8, 10–12).

**FIG 1:**
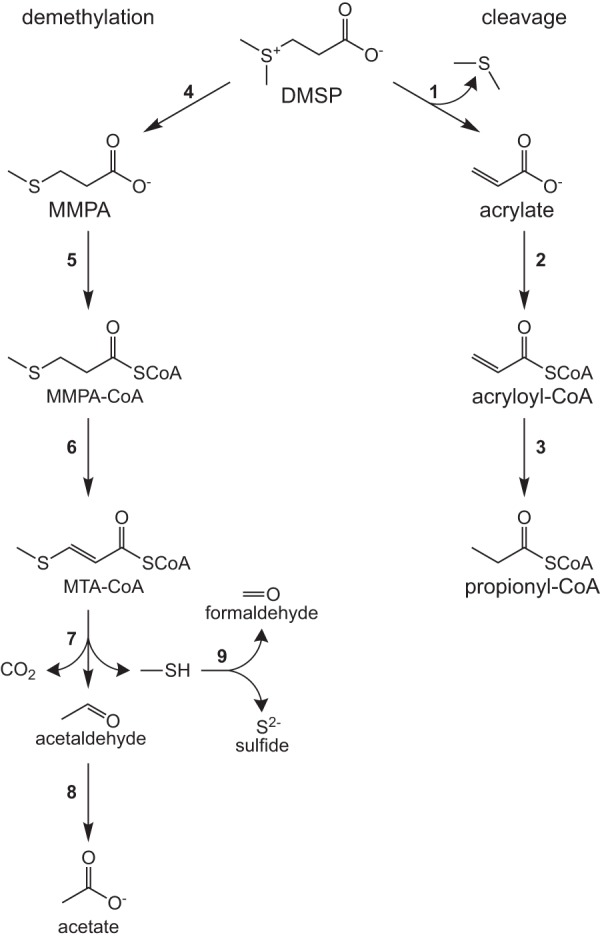
Metabolism of DMSP in Ruegeria pomeroyi DSS-3 and Ruegeria lacuscaerulensis ITI-1157. The two possible pathways for DMSP degradation are the cleavage pathway and the demethylation/demethiolation pathway. Propionyl-CoA formed in the cleavage pathway is further metabolized via the ethylmalonyl-CoA pathway (23). Abbreviations: MMPA, methylmercaptopropionic acid; MTA-CoA, methylthioacryloyl-CoA; SCoA, coenzyme A. Genes are indicated by the following numbers: 1, *dddD*, *dddP*, *dddQ*, and/or *dddW*; 2, *prpE*; 3, *acuI*; 4, *dmdA*; 5, *dmdB*; 6, *dmdC*; 7, *dmdD* and/or *acuH*; 8, *aldH*; 9, *mtoX*. The locus tags for each organism are provided in Table S1 in the supplemental material.

Bacterial catabolism of DMSP proceeds through one of three pathways. In the demethylation pathway, DMSP is demethylated to form methylmercaptopropionate (MMPA), which can be further broken down into methanethiol, carbon dioxide, and acetaldehyde (Fig. 1). Methanethiol can then be assimilated into biomass or broken down to formaldehyde and H_2_S. Alternatively, DMSP can undergo cleavage to form DMS and acrylate (2, 8, 13, 14). Lastly, DMSP can be oxidized to dimethylsulfoxonium propionate (DMSOP) by marine algae, which is then cleaved to dimethyl sulfoxide by bacterioplankton (15). While the genes encoding DMSOP metabolism are not known, the genes for the other pathways are widely conserved in marine bacterioplankton.

10.1128/mBio.00329-20.1 TABLE S1 DMSP genes in Ruegeria pomeroyi DSS-3 and Ruegeria lacuscaerulensis ITI-1157. Download Table S1, PDF file, 0.1 MB. Copyright © 2020 Wirth et al. 2020 Wirth et al. This content is distributed under the terms of the Creative Commons Attribution 4.0 International license.

There is substantial variation in the genes for DMSP metabolism in the roseobacter group (16). For instance, Ruegeria pomeroyi and Ruegeria lacuscaerulensis represent different clades within the genus *Ruegeria*, a member of the *Rhodobacteraceae* family of the class *Alphaproteobacteria* (17). Although they share many of the genes encoding DMSP metabolism, there are some differences (see Table S1 in the supplemental material) (16). Both *R. pomeroyi* and *R. lacuscaerulensis* possess *dmdA*, which encodes the first step of the demethylation pathway, as well as two homologues of *dmdB* and *dmdC*, which encode the next two steps. However, *R. pomeroyi* also possesses a third homologue of *dmdC*, the gene for a highly specific hydratase, *dmdD*, and a methanethiol oxidase gene, or *mtoX*, all of which are absent in *R. lacuscaerulensis*. In contrast, *R. lacuscaerulensis* apparently uses a multifunctional hydratase encoded by the gene *acuH* to catalyze the same reaction as DmdD (18). While *R. lacuscaerulensis* also lacks a homologue for *mtoX*, it possesses two genes in the same selenium-binding protein family that may catalyze this reaction. *R. pomeroyi* has four different DMSP lyase genes, but *R. lacuscaerulensis* has only two. Lastly, the DMSP transporter has not been identified in either bacterium, so that is another potential difference. The differences in gene content are apparently reflected in growth properties. For instance, *R. lacuscaerulensis* grows much more slowly than *R. pomeroyi* on DMSP as the sole carbon source and produces much less DMS and methanethiol (18).

The fate of DMSP in microbial biomass is not well understood (6, 19–23). During incubations of seawater cultures with [^35^S]DMSP, approximately 40% of the provided ^35^S was taken up by cells, with nearly 60% of this contained in trichloroacetic acid (TCA)-insoluble material. In addition, approximately 45% of the provided [^35^S]methanethiol was taken up by cells, and nearly 90% of this was present in TCA-insoluble material (19). To investigate this further, Kiene et al. (19) examined the metabolism of DMSP and methanethiol in pure cultures of several species. All tested species could efficiently incorporate ^35^S from labeled methanethiol into TCA-insoluble material, but only species capable of producing methanethiol from DMSP were capable of efficiently incorporating ^35^S from labeled DMSP (19). Finally, seawater cultures fed [*methyl*-^3^H]methanethiol incorporated a large portion of the radioisotope into methionine (19). Based on these results, the authors concluded that it was likely that methanethiol was directly incorporated into methionine via a reaction catalyzed by cystathionine γ-synthetase and that it was unlikely that methanethiol was oxidized to sulfide and formaldehyde before incorporation into methionine.

This hypothesis was supported in a second study that found that a mutant of Corynebacterium glutamicum lacking the 5,10-methylene-tetrahydrofolate (methylene-THF) reductase gene (*metF*) could not grow on sulfate as the sole sulfur source but that it could grow on sulfate plus methanethiol (20). Furthermore, when the C. glutamicum Δ*metF* mutant was grown on 99% [^13^C_6_]glucose and unlabeled methanethiol, 95% of the resulting methionine was unlabeled at the methyl carbon, indicating that nearly all of the methionine in this mutant was synthesized via the direct capture of methanethiol (20).

**FIG 2:**
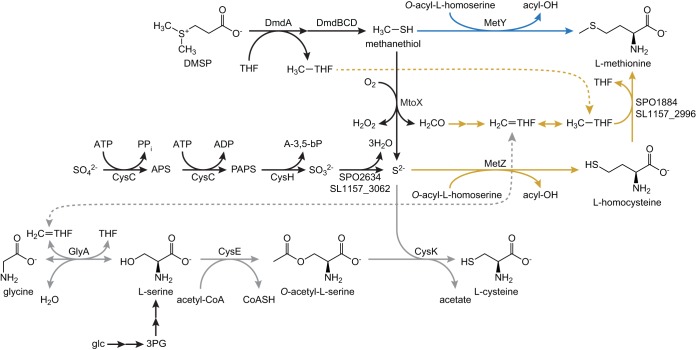
Putative pathways for the biosynthesis of l-methionine and l-cysteine during growth on glucose plus DMSP. Both possible routes for synthesizing l-methionine are shown, with the direct capture pathway highlighted in blue and the reassembly pathway highlighted in gold. The pathway for l-cysteine biosynthesis is highlighted in gray. Black arrows indicate reactions that are used in multiple pathways. Dashed lines connect common intermediates found in the THF pool. Abbreviations: glc, glucose; 3PG, 3-phosphoglycerate; APS, adenosine-5′-phosphosulfate; PAPS, 3′-phosphoadenosine-5′-phosphosulfate; A-3,5-bP, adenosine-3′,5′-bisphosphate. Pathways are manually curated from the KEGG annotations. Homologues of all proteins depicted are present in both *R. pomeroyi* and *R. lacuscaerulensis*, with the exception of MtoX, which is absent in *R. lacuscaerulensis*.

Further examination of the fate of isotopically labeled atoms from either di(methyl-^13^C)sulfoniopropionate ([*methyl*-^13^C]DMSP) or dimethylsulfoniopropionate-1-^13^C ([1-^13^C]DMSP) in *R. pomeroyi* found that the methyl carbon of l-methionine and the C-3 position of l-serine were enriched by 99% and 30%, respectively, following growth with [*methyl*-^13^C]DMSP as the sole carbon source (23). However, when *R. pomeroyi* was grown with [1-^13^C]DMSP as the sole carbon source, l-methionine was not enriched (23). Taken together, these data demonstrated that the methyl carbon of l-methionine was derived from the methyl carbons of DMSP and ruled out the possibility that methylmercaptopropionate (Fig. 1) was converted directly to l-methionine via reductive carboxylation and transamination. Presumably, the labeling observed at the C-3 position of l-serine when *R. pomeroyi* was grown on [*methyl*-^13^C]DMSP was due to its synthesis in part via l-glycine and methylene-THF or an exchange reaction catalyzed by serine hydroxymethyltransferase (Fig. 2).

**FIG 3:**
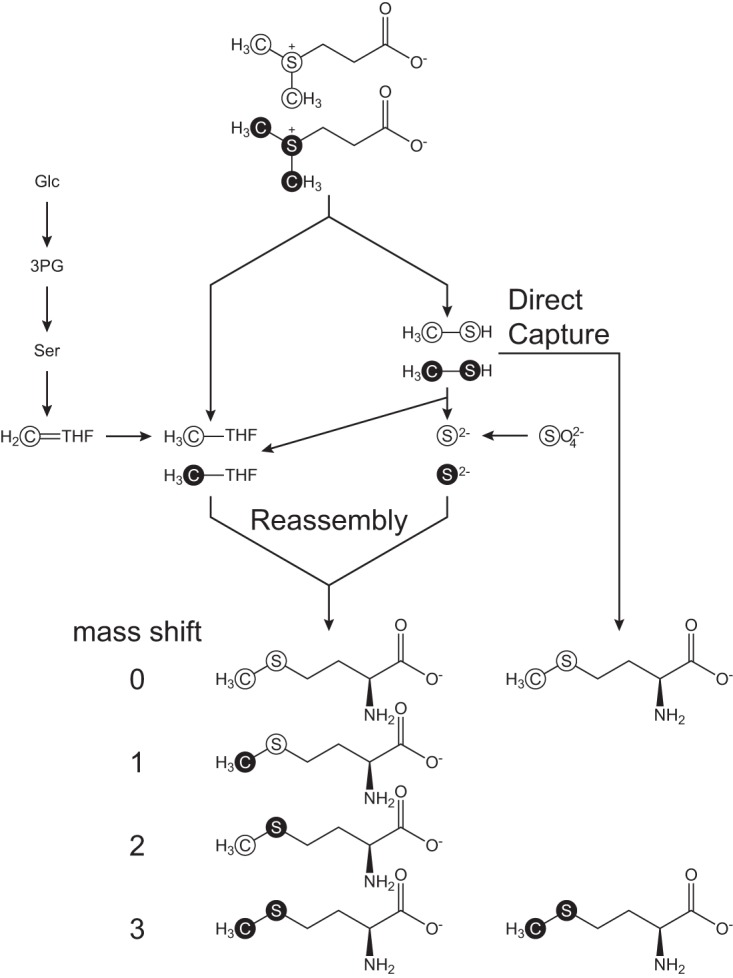
Labeling strategy for distinguishing the pathway for l-methionine biosynthesis from a mixture of labeled and unlabeled DMSP. Both the direct capture and reassembly pathways result in l-methionine that is either unenriched (mass shift of 0) or enriched with both ^13^C and ^34^S (mass shift of 3). However, the reassembly pathway also produces l-methionine that is enriched in either ^13^C or ^34^S, i.e., singly labeled (mass shifts of 1 and 2, respectively). Filled circles indicate atoms that are enriched with either ^13^C or ^34^S. Open circles indicate atoms with the natural abundance of isotopes. Abbreviations: Glc, glucose; 3PG, 3-phosphoglycerate; Ser, l-serine; THF, tetrahydrofolate.

However, these results failed to distinguish between the two alternatives for the biosynthesis of methionine from DMSP. In the first possibility, methanethiol is converted to l-methionine directly via a γ-substitution with *O*-acyl-l-homoserine (“direct capture” pathway), as previously suggested by Kiene et al. (19) (Fig. 2, blue lines). Alternatively, methanethiol could first be oxidized to formaldehyde and sulfide. The resulting sulfide could be converted to l-homocysteine via a γ-substitution with *O*-acyl-l-homoserine. Formaldehyde could be oxidized to formate by formaldehyde dehydrogenase, leading to the formation of formyl-THF. Alternatively, formaldehyde could react chemically with THF to form methylene-THF. In either case, these compounds could then be reduced to methyl-THF for the methylation of l-homocysteine to produce methionine (“reassembly” pathway) (Fig. 2, gold lines). In order to investigate which pathways were being used for methionine biosynthesis, *R. pomeroyi* or *R. lacuscaerulensis* was grown with a 1:1 mixture of di(*methyl*-^13^C)sulfonio-^34^S-propionate ([^13^C, ^34^S]DMSP) and unlabeled DMSP. Analyses of the resulting l-methionine and l-cysteine isotopomers made it possible to distinguish which pathways were being utilized (Fig. 3). For example, if all of the l-methionine was synthesized via the “direct capture” pathway, then the resulting l-methionine would be either unenriched (mass shift of 0) or enriched with both ^13^C and ^34^S (mass shift of 3). However, if all of the l-methionine was synthesized via the “reassembly” pathway, then there would be a mixture of unenriched l-methionine (mass shift of 0) and l-methionine enriched with ^13^C only (mass shift of 1), ^34^S only (mass shift of 2), and both ^13^C and ^34^S (mass shift of 3) (Fig. 3). If both pathways were used, then the fraction of l-methionine synthesized by each pathway could be determined from the relative enrichments of the l-methionine isotopomers.

Furthermore, examination of the enrichment of l-cysteine provided additional information about the reduced sulfur and carbon pools. For instance, the ^34^S enrichment of l-cysteine provided a measure of the intracellular sulfide pools. Two processes affected the ^13^C enrichment of cysteine. Cysteine was biosynthesized from serine, and unlabeled serine was derived largely from unlabeled glucose via 3-phosphoglycerate. Then, an exchange reaction catalyzed by serine hydroxymethyltransferase transferred label from methylene-THF into the C-3 of serine. Thus, the ^13^C enrichment of cysteine provided a measure of the enrichment of the methylene-THF pool. For comparison, the enrichment of the methyl-THF pool could be obtained from the enrichment of the methionine formed by the reassembly pathway.

## RESULTS

### DMSP utilization differs in Ruegeria lacuscaerulensis and Ruegeria pomeroyi.

**FIG 4:**
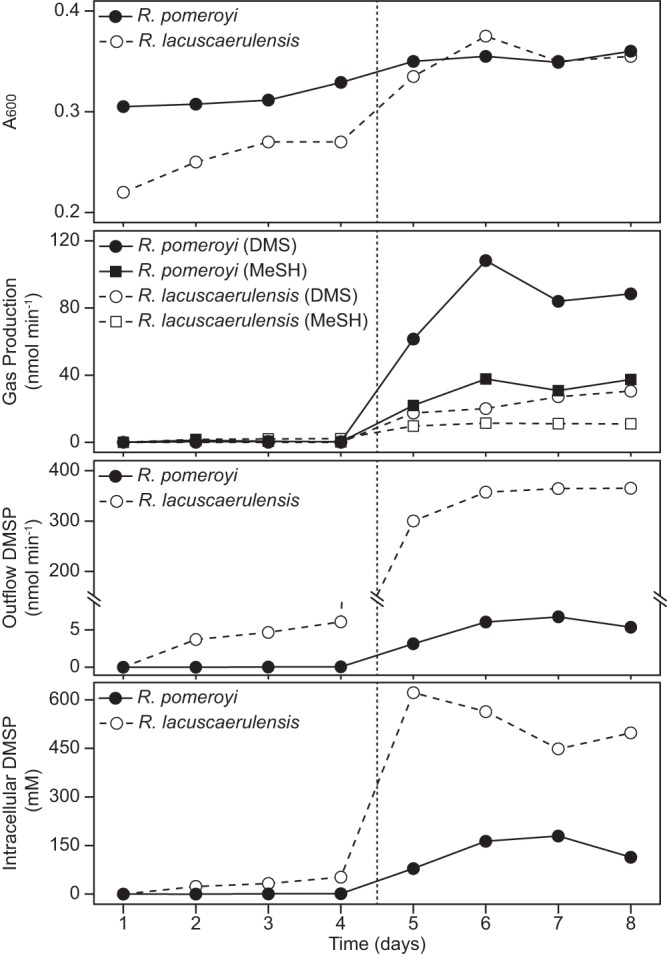
Metabolism of dimethylsulfoniopropionate by *R. pomeroyi* and *R. lacuscaerulensis* chemostat cultures. Cultures were grown on minimal medium supplemented with 2 mM glucose. At time zero, DMSP in the reservoir was increased to 100 μM for an addition rate of 10 nmol min^−1^. After 4 days, the DMSP in the reservoir was increased to 5 mM for an addition rate of 500 nmol min^−1^ (indicated by the broken vertical line). The scale of the *y* axis for the outflow DMSP plot has been broken (diagonal lines) to show both the low and high values. Additional measurements are reported in Table 1.

*R. pomeroyi* and *R. lacuscaerulensis* were grown in chemostats to compare their DMSP utilization at either low or high levels (Fig. 4). In these experiments, glucose was present in the medium at 2 mM, and it was consumed to very low levels of <2 μM. Although the growth yields of *R. pomeroyi* and *R. lacuscaerulensis* were similar, the amounts of DMSP metabolized and gases produced differed. In a typical chemostat culture fed 100 μM DMSP, *R. pomeroyi* consumed most of the DMSP and reduced the concentration in the chemostat to 0.3 μM (Table 1). In contrast, under the same conditions, *R. lacuscaerulensis* consumed just over 50% of the DMSP, and the concentration remaining in the chemostat was about 48 μM. About 4% and 7% of the DMSP fed to *R. pomeroyi* were released as DMS and methanethiol, respectively. In contrast, the DMS and methanethiol released by *R. lacuscaerulensis* were <1% and 20% of the DMSP, respectively. When fed 5 mM DMSP, *R. pomeroyi* again consumed almost 99% and released about 19% and 7% as DMS and methanethiol, respectively (Table 1). In contrast, *R. lacuscaerulensis* consumed only 37% and released only 5% and 2% as DMS and methanethiol, respectively. An especially dramatic difference was the high levels of intracellular DMSP in *R. lacuscaerulensis*, 36 and 503 mM, during growth on low and high levels of DMSP, respectively, compared to 1 and 84 mM in *R. pomeroyi.* These results implied that these two species metabolized DMSP very differently. Under these conditions, dimethyldisulfide production was not detected in either culture (data not shown). Although not measured in these experiments, only low levels of dimethyl sulfoxide (DMSO) have been detected in other experiments with these bacteria. In fact, oxidation of DMS to DMSO has been shown to be dependent on the presence of trimethylamine in *R. pomeroyi*, as this is accomplished by a trimethylamine monooxygenase (SPO1551) (24). There is no homologue of this protein in *R. lacuscaerulensis*.

**TABLE 1:** DMSP consumption and metabolic demands of *R. pomeroyi* and *R. lacuscaerulensis* chemostat cultures

Parameter	Value^*a*^ for:			
	*R. pomeroyi*		*R. lacuscaerulensis*	
Inflow DMSP	10	500	10	500
DMS produced	0.41 ± 0.03	93.6 ± 12.3	0.01 ± 0.00	25.9 ± 7.6
MeSH produced	0.70 ± 0.51	35.3 ± 4.0	1.99 ± 0.28	11.2 ± 0.9
Outflow DMSP	0.03 ± 0.03	6.06 ± 1.63	4.82 ± 1.12	362.4 ± 5.7
CH_3_ (DMSP) consumed^*b*^	9.56 ± 0.06	400.4 ± 12.5	5.16 ± 1.12	111.7 ± 9.2
MeSH consumed^*c*^	8.85 ± 0.47	365.0 ± 15.4	3.18 ± 1.33	100.6 ± 8.9
S demand^*d*^	3.43 ± 0.12	3.86 ± 0.13	2.85 ± 0.17	3.92 ± 0.25
C-1 demand^*d*^	15.6 ± 0.6	17.5 ± 0.6	12.9 ± 0.8	17.7 ± 1.1
Biomass C^*e*^	425 ± 15	478 ± 16	353 ± 22	485 ± 31

a Unless stated otherwise, all values are reported in nanomoles per minute and are the means (*n *= 6) of results for days 2 through 4 after the addition of either 100 μM DMSP or 5 mM DMSP to the chemostat reservoir. The error indicates the 95% confidence intervals. Abbreviations: DMSP, dimethylsulfoniopropionate; DMS, dimethylsulfide; MeSH, methanethiol; C-1, reduced C-1.

b Amount of CH_3_ C consumed by the initial demethylation of DMSP. Values were calculated by subtracting the rates of DMS production and outflow DMSP from the rate of inflow DMSP.

c Amount of both the CH_3_ C consumed by the assimilation of methanethiol by either the direct capture or reassembly pathways and the total S consumed. Values were calculated by subtracting the rates of DMS and MeSH gas production and outflow DMSP from the rate of inflow DMSP.

d The reduced carbon and sulfur demands were calculated from the cell yields as described in Materials and Methods.

e The cellular carbon was calculated as described in Materials and Methods.

### Sulfur and methyl C assimilation by Ruegeria pomeroyi.

To examine the DMSP metabolism in greater detail, chemostat cultures were fed 100 μM DMSP (1:1 DMSP to [^13^C, ^34^S]DMSP) at a rate of 10 nmol min^−1^ in minimal medium with 2 mM glucose. The measured specific enrichment of [^13^C, ^34^S]DMSP in the medium was 50.8% ± 0.3%, or very close to the expected level. In this experiment, the concentration of DMSP in the culture outflow was 0.8 ± 0.1 μM, and the rates of DMS and methanethiol production were 0.35 ± 0.02 and 0.13 ± 0.04 nmol min^−1^, respectively (Table 2). These values were similar to those obtained in experiments with unenriched DMSP (Table 1), and the differences were typical of replicate chemostat experiments. Under these conditions, the amount of DMSP sulfur consumed was about three times the anabolic demand for sulfur. DMSP can donate up to two carbons for biosynthesis via the THF pathway (Fig. 2). Carbon can be assimilated from methanethiol at either the formyl or methylene level depending upon the pathway of formaldehyde metabolism and at the methyl level from DMSP demethylation (Table 2). Because *R. pomeroyi* contains the enzymes to readily interconvert the THF derivatives, we refer to them collectively as C-1-THF. Based upon the amount of biomass formed, the C-1-THF demand for biosynthesis was 13.3 nmol min^−1^, or somewhat lower than 19.0 nmol min^−1^, the total amount of C-1-THF potentially available from DMSP (Table 2). Thus, C-1-THF oxidation must be concurrent with its assimilation under these conditions.

**TABLE 2:** DMSP consumption and metabolic demands of *R. pomeroyi* chemostat cultures during the labeling experiment

Parameter	Value for *R. pomeroyi*^*a*^
Inflow DMSP	10
DMS produced	0.35 ± 0.02
MeSH produced	0.13 ± 0.04
Outflow DMSP	0.08 ± 0.01
CH_3_ (DMSP) consumed^*b*^	9.57 ± 0.02
MeSH consumed^*c*^	9.43 ± 0.04
S demand^*d*^	3.28 ± 0.21
C-1 demand^*d*^	13.3 ± 1.4
Biomass C^*e*^	410 ± 20

a Unless stated otherwise, all values are reported in nanomoles per minute and are the means (*n *= 10) of results for days 2 through 6 after the addition of 100 μM DMSP (50 μM unenriched DMSP and 50 μM [^13^C, ^34^S]DMSP) to the chemostat reservoir. The error indicates the 95% confidence intervals. Abbreviations: DMSP, dimethylsulfoniopropionate; DMS, dimethylsulfide; MeSH, methanethiol; C-1, reduced C-1.

b Amount of CH_3_ consumed by the demethylation of DMSP. Values were calculated by subtracting the rates of DMS production and outflow DMSP from the rate of inflow DMSP.

c Amount of both the CH_3_ consumed by the assimilation of methanethiol by either the direct capture or reassembly pathway and the total S consumed. Values were calculated by subtracting the rates of DMS and MeSH production and outflow DMSP from the rate of inflow DMSP.

d The reduced carbon and sulfur demands were calculated from the cell yields as described in Materials and Methods.

e Values are the means (*n *= 5) of results for days 2 through 6 after the addition of 100 μM DMSP (50 μM unenriched DMSP and 50 μM [^13^C, ^34^S]DMSP) to the chemostat reservoir. The cellular carbon was calculated as described in Materials and Methods.

l-Cysteine was expected to be synthesized from the sulfide and l-serine pools. The enrichments of ^34^S-containing l-cysteine isotopomers indicated that DMSP was the major source of sulfur for l-cysteine biosynthesis. On average, the ^34^S enrichment of l-cysteine was 47.7% ± 1.1%, or very close to the enrichment of [^13^C, ^34^S]DMSP in the medium (Fig. 5C). In contrast, the ^13^C enrichment of l-cysteine was 13.6% ± 3.6%, or much less than the enrichment of [^13^C, ^34^S]DMSP in the medium (Fig. 5C). This result was consistent with the formation of unlabeled l-serine from glucose and the introduction of label from the C-1-THF pool by an exchange reaction catalyzed by the reversible enzyme l-serine hydroxymethyltransferase.

**FIG 5:**
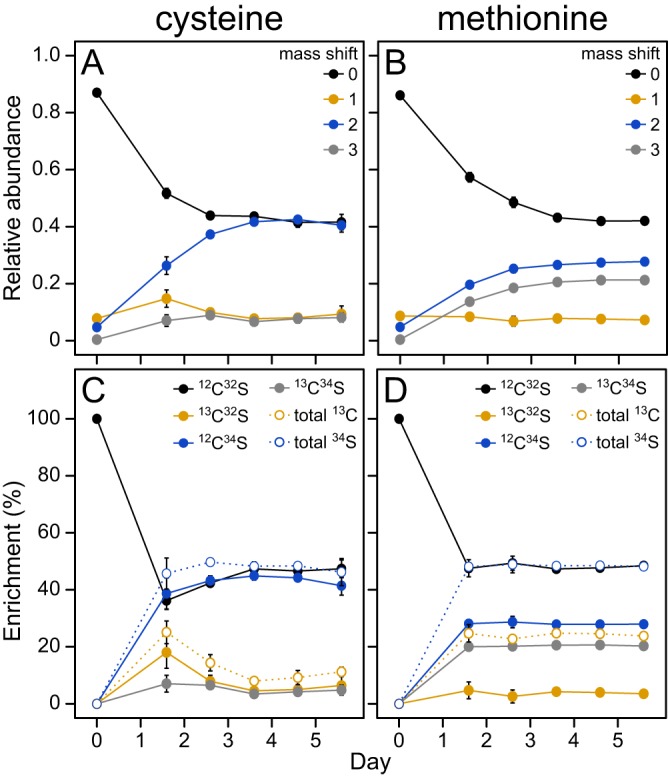
Labeling profile of l-methionine and l-cysteine in an *R. pomeroyi* chemostat culture. All plots depict the labeling of the cellular l-methionine and l-cysteine for the 5 days following the addition of 100 μM DMSP (50.8% enriched with [^13^C, ^34^S]DMSP). The percent enrichment of each isotopomer (C and D) was calculated by correcting the observed relative abundances (A and B) for the natural abundance of isotopes and then correcting for the presence of legacy material in the samples as described in Materials and Methods. Error bars indicate the 95% confidence intervals and are not shown when they are smaller than the symbols.

As was seen with l-cysteine, the ^34^S enrichment of l-methionine indicated that DMSP was the major sulfur source for l-methionine biosynthesis as well (Fig. 5D). However, the enrichment of ^13^C-containing l-methionine was much higher than that of l-cysteine. In principle, the high ^13^C enrichment could be due to either the direct capture or reassembly pathway if the C-1-THF pool was highly labeled. In fact, the extent of enrichment was, on average, consistent with the formation of 66.2% ± 1.5% of the l-methionine via the reassembly pathway (see below). Moreover, the portion of l-methionine made via reassembly did not differ greatly over the 5 days of the experiment, ranging between 62.8% ± 4.1% and 70.1% ± 6.1%, implying that the biosynthetic pathway did not change materially during this time.

### Specific enrichments of metabolic pools in Ruegeria pomeroyi.

**FIG 6:**
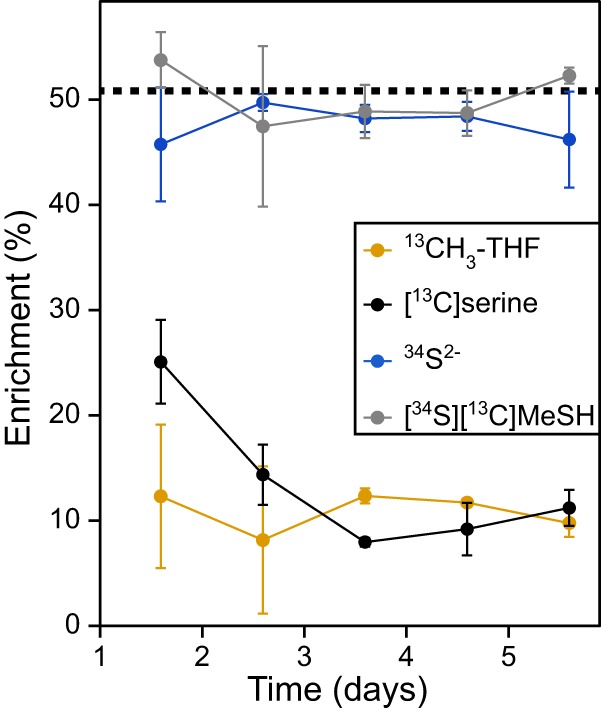
Specific enrichment of the molecules derived from methyl-THF, l-serine, sulfide, and methanethiol in an *R. pomeroyi* chemostat culture. The percentage of each pool that was enriched following the addition of 100 μM DMSP (50.8% enriched with [^13^C, ^34^S]DMSP) is shown. The dashed line indicates the percentage of [^13^C, ^34^S]DMSP, and the thickness of this line indicates its 95% confidence interval. The enrichments were calculated from the data in Fig. 5 as described in Materials and Methods.

The enrichment of the sulfide and methyl-THF pools provided additional insights into DMSP metabolism in *R. pomeroyi*. The enrichment of the methyl-THF, l-serine, sulfide, and methanethiol pools (Fig. 6) were calculated from the legacy-corrected enrichments (Fig. 5C and D). On average, ^13^C enrichment in the CH_3_-THF and l-serine pools were 10.9% ± 1.3% and 13.6% ± 3.6%, respectively, and not significantly different. This result suggested that the exchange reactions between the C-1-THF intermediates were rapid enough to bring them into isotopic equilibrium. ^34^S enrichment in the sulfide pool was 47.7% ± 1.0%, and the enrichment of methanethiol with both ^13^C and ^34^S was 50.2% ± 1.6%. Thus, DMSP accounted for nearly all of the sulfide pool during steady-state growth. Even after only 38 h, or the first sampling following the introduction of DMSP to the culture, 90.0% ± 11.8% of the sulfide (or about twice the observed value of 45.7%) in the nonlegacy material could be attributed to DMSP (Fig. 6). Thus, assimilatory sulfate reduction was rapidly shut down in the presence of DMSP. Lastly, because the amount of DMSP-derived sulfur consumed by the cell was nearly three times the estimated sulfur demand, sulfur assimilation must have been accompanied by large amounts of sulfur oxidation (Table 2).

### Incorporation of DMSP by Ruegeria pomeroyi and Ruegeria lacuscaerulensis.

**FIG 7:**
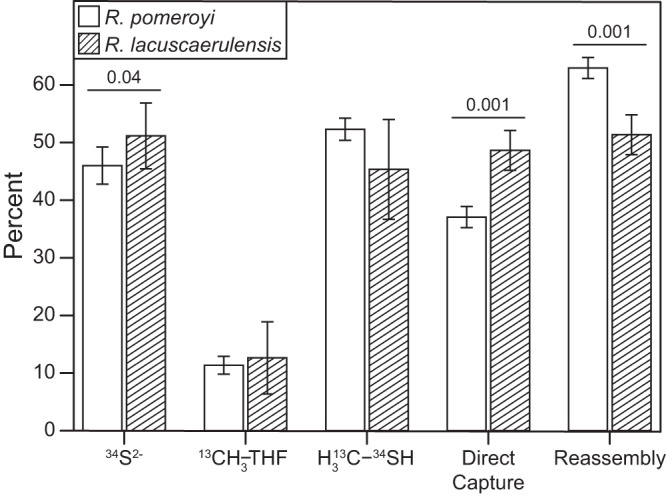
Comparison of labeling by [^13^C, ^34^S]DMSP in *R. pomeroyi* and *R. lacuscaerulensis* chemostat cultures. Bars indicate either the percentage of each pool that was labeled after 5 days following the addition of 50 μM DMSP (50.18% ± 2.24% enriched with [^13^C, ^34^S]DMSP) or the percentages of l-methionine synthesized via the respective pathways (*n *= 3). Error bars indicate the 95% confidence intervals. *P* values of <0.05 are shown above each comparison.

In a similar labeling experiment, *R. pomeroyi* and *R. lacuscaerulensis* were grown in chemostats to compare their utilization of DMSP. Because their metabolisms were strikingly different, it was hypothesized that the pathways for assimilation of DMSP methyl C and S would also differ. On the contrary, the specific enrichments of the metabolic pools for the l-methionine precursors were strikingly similar (Fig. 7; see Tables S2 to S4 in the supplemental material). The [^13^C]methyl-THF and doubly enriched methanethiol pools were not significantly different (Table S4). Although the ^34^S-enriched sulfide pools were statistically different, their values were similar. For instance, the sulfide pools of *R. pomeroyi* and *R. lacuscaerulensis* were 46.0% ± 3.2% and 51.2% ± 5.7% enriched with ^34^S, respectively (Fig. 7). Moreover, the relative utilizations of the two l-methionine biosynthetic pathways were similar. For instance, the percentages of l-methionine synthesized via the reassembly pathway by *R. pomeroyi* and *R. lacuscaerulensis* were 62.9% ± 1.8% and 51.4% ± 3.4%, respectively (Fig. 7). These results suggested that although the catabolism of DMSP differed between *R. pomeroyi* and *R. lacuscaerulensis*, the assimilation of DMSP methyl C and S was similar.

10.1128/mBio.00329-20.2 TABLE S2 Comparison of the relative abundances of mass shifts for *R. pomeroyi* and *R. lacuscaerulensis* chemostat cultures. Download Table S2, PDF file, 0.1 MB. Copyright © 2020 Wirth et al. 2020 Wirth et al. This content is distributed under the terms of the Creative Commons Attribution 4.0 International license.

10.1128/mBio.00329-20.3 TABLE S3 Comparison of the isotopomer enrichments for *R. pomeroyi* and *R. lacuscaerulensis* chemostat cultures. Download Table S3, PDF file, 0.1 MB. Copyright © 2020 Wirth et al. 2020 Wirth et al. This content is distributed under the terms of the Creative Commons Attribution 4.0 International license.

10.1128/mBio.00329-20.4 TABLE S4 Comparison of the enriched pools and pathway fluxes for *R. pomeroyi* and *R. lacuscaerulensis* chemostat cultures. Download Table S4, PDF file, 0.1 MB. Copyright © 2020 Wirth et al. 2020 Wirth et al. This content is distributed under the terms of the Creative Commons Attribution 4.0 International license.

## DISCUSSION

Previously, it was demonstrated that only roseobacter strains which could metabolize DMSP to methanethiol were able to incorporate significant amounts of ^35^S from [^35^S]DMSP into TCA-insoluble material (19). This finding, along with others, led to the hypothesis that the majority of methionine biosynthesis in the roseobacter group was accomplished by the direct capture of methanethiol (19, 20, 23, 25, 26). Although the majority of l-methionine sulfur in *R. pomeroyi* DSS-3 was derived from DMSP, only about one-third was synthesized via the direct capture pathway. This result did not disagree with previous experiments examining DMSP metabolism in *R. pomeroyi* but refuted the interpretation that the direct capture of methanethiol is the major source of l-methionine during growth with DMSP. Moreover, it demonstrated that even at the low concentrations of <1 μM present in the chemostat, DMSP was the major sulfur source for *R. pomeroyi* despite the presence of >14 mM sulfate in the medium. Similarly, DMSP was the major sulfur source for *R. lacuscaerulensis* even though only a portion was taken up or metabolized to DMS or methanethiol.

The metabolic differences between *R. pomeroyi* and *R. lacuscaerulensis* are likely due to a number of factors, including the gene content and properties of their enzymes. For instance, the DmdB homologues in *R. pomeroyi* have high affinities for MMPA, with one homologue having a slightly higher affinity for crotonate (27). In contrast, the two DmdB homologues in *R. lacuscaerulensis* have significantly higher affinities for either acetate, propionate, valerate, or butyrate (27). In addition, DmdB in both *R. pomeroyi* and *R. lacuscaerulensis* is strongly inhibited by DMSP. In *R. pomeroyi*, this inhibition is reversed by the presence of either ADP or MMPA, but the DmdB homologues in *R. lacuscaerulensis* remain inhibited even at high concentrations of ADP or MMPA (27). Therefore, the *R. lacuscaerulensis* enzymes are expected to possess low activity under conditions where DMSP accumulates intracellularly. Likewise, *R. pomeroyi* possesses a hydratase (DmdD) with high activity toward its substrate, methylthioacrylyl coenzyme A (methylthioacrylyl-CoA), while *R. lacuscaerulensis* appears to utilize a multifunctional enzyme (AcuH) for the same reaction (18). These reactions result in the production of methanethiol, which can be oxidized to sulfide, formaldehyde, and hydrogen peroxide by methanethiol oxidase (MtoX) (Fig. 1). The lack of a homologue for MtoX in *R. lacuscaerulensis* presents a problem for the cell, as methanethiol can be toxic. However, the enrichments indicated that 51.4% ± 3.4% of the methionine synthesized by *R. lacuscaerulensis* was accomplished via the reassembly pathway, which suggests that methanethiol is converted to sulfide via another mechanism (see Table S4 in the supplemental material). Previously, it was demonstrated that an *R. pomeroyi* strain lacking *mtoX* lost more than 90% of its methanethiol oxidase activity, but the agent responsible for the remaining activity has not yet been identified (28). MtoX is a member of the selenium-binding protein family; there are two additional members of this family with low but significant sequence similarity to MtoX in *R. pomeroyi*, and homologues to these proteins are also present in *R. lacuscaerulensis*. It is possible that these proteins participate in the oxidation of methanethiol in *R. lacuscaerulensis*, but only a few studies have characterized other methanethiol oxidases at the genetic and biochemical levels (29). Furthermore, the absence of MtoX in *R. lacuscaerulensis* may explain why it has a higher percentage of methionine synthesized via the direct capture pathway than *R. pomeroyi* (Fig. 7).

*R. pomeroyi* possesses four DMSP cleavage enzymes, while *R. lacuscaerulensis* has only two (Table S1). All of these enzymes produce acrylate and DMS from DMSP, with the exception of DddD, which produces 3-hydroxypropionate and DMS (2, 23). The acrylate is first converted to acryloyl-CoA by PrpE, which can then either be reduced to propionyl-CoA by AcuI or converted to 3-hydroxypropionyl-CoA by AcuH (2, 23). Like methanethiol, acrylate is a reactive compound that can cause cellular damage if allowed to accumulate in the cell. In both *R. pomeroyi* and *R. lacuscaerulensis*, homologues are present for the proteins involved in acrylate metabolism, namely, PrpE, AcuH, and AcuI (Table S1). The additional DMSP cleavage proteins may explain why *R. pomeroyi* produces more DMS per mol DMSP than *R. lacuscaerulensis* (Table 1). In particular, the presence of DddD allows *R. pomeroyi* to produce 3-hydroxypropionate directly from DMSP, which may in turn enable a higher rate of DMSP cleavage, as this reaction would avoid the production of acrylate.

Although closely related, these two species appear to have adapted to very different lifestyles. Based upon their exoprotein secretion strategies, *R. pomeroyi* manipulates other members of its community with toxin-like compounds (30). In contrast, the exoproteome of *R. lacuscaerulensis* is heavily weighted toward transporters, suggesting that nutrient uptake is an important adaptation to its lifestyle. In spite of the similarity in gene content, DMSP metabolism must be integrated into functionally very different organisms, resulting in differences in the way that DMSP is utilized. However, the enzymatic properties of DMSP metabolism are not fully understood, and other factors may contribute to the observed differences (8, 14). These differences may confound ecological studies which aim to predict functional properties of microbial communities from the presence or absence of specific genes. For instance, the presence of *dmdA*, the first gene in the pathway, is often used to imply the presence of an active demethylation pathway (31, 32). However, the presence of this gene is inherently ambiguous. Both *R. pomeroyi* and *R. lacuscaerulensis* possess *dmdA*, but they use DMSP very differently.

In order for *R. pomeroyi* and *R. lacuscaerulensis* to synthesize l-cysteine from DMSP, they must first convert methanethiol to sulfide, which may explain why these organisms synthesize much of their l-methionine via the reassembly pathway. Although the direct capture of methanethiol is more efficient for the biosynthesis of l-methionine, it competes directly with the formation of sulfide for the biosynthesis of l-cysteine and other sulfur compounds. Moreover, starting with *O*-acyl-l-homoserine, the reassembly pathway requires only two more pairs of electrons than the direct capture pathway and is nearly energy equivalent. Lastly, both *R. pomeroyi* and *R. lacuscaerulensis* possess an l-methionine γ-lyase (MegL) which is capable of producing methanethiol from l-methionine (data not shown) and initiating a futile cycle when l-methionine is abundant. Therefore, cells must carefully regulate the levels of intracellular l-methionine to avoid this wasteful process.

## MATERIALS AND METHODS

### General.

All glassware used in the amino acid extractions and subsequent derivatizations (see below) was acid washed in 3% (vol/vol) HCl for 24 h to remove trace contaminants and then baked at 180°C for 24 h to degrade any remaining organic compounds. Dry HCl was generated as previously described (33). Methanolic HCl was generated by bubbling dry HCl into methanol while stirring. The solution was titrated to determine the concentration of HCl and stored in a stoppered glass bottle under an atmosphere of nitrogen at −20°C for no more than 1 month.

### Synthesis of substrates.

Di(*methyl*-^13^C)sulfonio-^34^S-propionate ([^13^C, ^34^S]DMSP) hydrochloride was synthesized as previously described (33). Briefly, ^34^S_8_ was reduced to Na_2_^34^S via a Birch reduction in liquid ammonia (34, 35). The resulting Na_2_^34^S was converted to di(*methyl*-^13^C)sulfide-^34^S ([^13^C, ^34^S]DMS) via methylation with I^13^CH_3_ under alkaline conditions and purified via distillation (36). The purified [^13^C, ^34^S]DMS was then converted to [^13^C, ^34^S]DMSP hydrochloride via a Michael addition to acrylic acid and washed with CH_2_Cl_2_ to remove any excess reactants (37). The isotopic purity of the resulting compound was greater than 98% (33).

### Chemostat cultures.

*R. pomeroyi* DSS-3 was grown at 30°C on a carbon-limited chemostat as previously described with a minimal medium composed of 50% (vol/vol) general salts solution, 0.08 M HEPES (pH 6.8), 0.58 mM KH_2_PO_4_, 0.068 mM FeEDTA, 0.1% (vol/vol) trace mineral solution, and 0.1% (vol/vol) vitamin solution (38, 39). Briefly, the chemostat was inoculated with 1 ml of a culture of *R. pomeroyi* grown in 1/2 YTSS medium (DSMZ medium no. 974) at 30°C for 24 h. At this point, a pump connected to a reservoir containing minimal medium supplemented with 2 mM glucose was turned on, and the chemostat was allowed to fill to the maximum volume of 150 ml at a rate of 0.1 ml min^−1^. After 21 days of growth, 50 μM DMSP and 50 μM [^13^C, ^34^S]DMSP (100 μM total DMSP) was added to the reservoir. At 14.25 h after the addition of DMSP, the outflow was collected in a sterile bottle on ice for 24 h. After each 24-h interval, the cells were harvested via centrifugation at 6,000 × *g* for 30 min at 4°C. Pellets were washed once with 10 ml of distilled, deionized water and then stored at −80°C until processing. Dimethylsulfide (DMS) and methanethiol present in the headspace of the chemostats were measured twice per day. The chemostat was allowed to run for 5 days, at which point the contents of the chemostat were harvested.

The fraction of labeled to unlabeled DMSP in the sterile medium was determined as previously described (33). Briefly, 2 ml of sterile medium was transferred to a 10-ml serum vial. The vial was crimp sealed with a Teflon-coated, butyl rubber stopper, and the headspace atmosphere was replaced with nitrogen. A syringe was used to add 2 ml of 4 M NaOH to the vials, which were then vortexed, followed by incubation at 37°C for 2.5 h. The resulting DMS was analyzed via gas chromatography-mass spectrometry (GC-MS), and the relative abundances at *m/z* 62 (corresponding to the unlabeled DMS) and *m/z* 66 (corresponding to the [^13^C, ^34^S]DMS) were compared.

Another chemostat experiment was performed with cultures of either *R. pomeroyi* DSS-3 or *R. lacuscaerulensis* ITI-1157 as described above with two exceptions: 50 μM total DMSP (25 μM DMSP and 25 μM [^13^C, ^34^S]DMSP) were added to the chemostat instead of 100 μM total DMSP, and the chemostat outflows were collected immediately after the addition of DMSP to the medium reservoirs. All other conditions were identical to those described above.

A third chemostat experiment was performed with cultures of either *R. pomeroyi* DSS-3 or *R. lacuscaerulensis* ITI-1157 as described above with one exception: 100 μM unlabeled DMSP was added to the chemostat, this was increased to 5 mM DMSP after 4 days of growth, and the culture was allowed to grow for an additional 4 days. All other conditions were identical to those described above.

### Calculation of reduced carbon and sulfur demands and biomass.

The amount of dry weight was calculated from the *A*_660_ as previously described (40). The doubling time of the cultures (1,040 min) was calculated by dividing the natural log of 2 by the specific growth rate (μ) of the chemostat (6.67 × 10^−4 ^min^−1^) (41). For both the reduced carbon and sulfur demands, the values for each metabolite (micromoles per milligram [dry weight]) were assumed to be equal to those for Escherichia coli (42). The methylene plus methyl-tetrahydrofolate, or reduced C-1 carbon demand, was calculated as the sum of the amount of l-serine, l-methionine, dATP, dGTP, dTTP, ATP, and GTP required to sustain the dry weight divided by the doubling time. Because purines require two equivalents of reduced C-1 carbon, the values for dATP, dGTP, ATP, and GTP were doubled (43). The sulfur demand was calculated as the sum of the amount of l-cysteine and l-methionine required to sustain the dry weight divided by the doubling time. For biomass C, the concentration of dry weight as determined from the *A*_660_ was multiplied by the flow rate of the chemostat (0.1 ml min^−1^), and 50% of this dry weight was assumed to be carbon (44).

### Measurement of DMS and methanethiol.

Dimethylsulfide (DMS) and methanethiol were measured as previously described (23). Briefly, a gas-tight syringe was used to inject 1 ml of the headspace contents onto an SRI-8610-C gas chromatograph with a Chromosil 330 column with nitrogen carrier gas at a flow rate of 60 ml min^−1^, an oven temperature of 60°C, and a flame photometric detector. Under these conditions, DMS and methanethiol had retention times of approximately 1.60 min and 1.03 min, respectively. Standard curves generated from known amounts of DMS and methanethiol were used to convert peak areas to amounts of DMS and methanethiol in the gas phase. The DMS and methanethiol concentrations in the aqueous phase were then calculated by using the distribution coefficient for 10 ppm DMS or methanethiol at 30°C in artificial seawater (45).

### Amino acid extraction.

Amino acids were extracted and prepared for derivatization as previously described (46, 47). Cell pellets were thawed on ice and resuspended in 2 ml of a solution of 6 M urea dissolved in 0.5 M Tris, pH 8.6 (lysis buffer), and then incubated at −80°C for 20 min. The suspension was thawed and then lysed via four passages through a cell disruptor (One Shot; Constant Systems Ltd.) at 14.5 × 10^3^ lb/in^2^ (approximately 100,000 kPa) (13). The cell disruptor was then washed with 1 ml of lysis buffer and again with 2 ml of lysis buffer. The lysate and both washes were combined and transferred to a glass serum vial, which was then crimp sealed with a Teflon-coated butyl rubber stopper. The headspace atmosphere of the vial was replaced with nitrogen gas, and 500 μl of a freshly prepared, filter-sterilized, aqueous solution of 0.2 M dithiothreitol was added with a syringe. The vial was then incubated at 100°C for 10 min to reduce all cystinyl residues to thiols. The vial was cooled, and 100 μl of ICH_3_ was added with a syringe. The vial was incubated at 60°C with shaking at 300 rpm for 30 min. The contents of the vial were transferred to Spectra/Por 7 pretreated regenerated cellulose dialysis tubing with a 2,000 molecular weight cutoff (Spectrum Laboratories, Inc.; product no. 132107) and dialyzed twice against 1 liter of distilled, deionized water for 12 h at 4°C for a total of 24 h. The dialyzed cell lysate was transferred to a Balch tube, and the liquid was evaporated with a stream of nitrogen at 50°C. The tube was crimp sealed with a butyl rubber stopper, and the headspace atmosphere was replaced with nitrogen gas. A syringe was used to add 2 ml of an anaerobic 6 M HCl solution, and proteins were converted to free amino acids by incubating the tube at ≥110°C for 24 h. The tube was cooled to room temperature, and the liquid was evaporated with a stream of nitrogen at 50°C. The solids were dissolved in 1 ml of distilled, deionized water, and the liquid was transferred to a 1.5-ml microcentrifuge tube. The tube was centrifuged at 17,000 × *g* for 10 min to pellet the black precipitate, and the supernatant was transferred to a clean glass serum vial. The liquid was evaporated with a stream of nitrogen at 50°C, leaving behind solids composed primarily of amino acid hydrochloride salts and other water-soluble cellular debris.

### Derivatization of amino acids.

Amino acids were converted to their *N*-trifluoroacetyl amino acid methyl esters as previously described (46–48). Briefly, the vial containing the amino acid hydrochloride salts was crimp sealed with a butyl rubber stopper, and the headspace atmosphere was replaced with nitrogen gas. A syringe was used to add 1 ml of freshly prepared 4 M methanolic HCl. The vial was incubated in a boiling water bath for 30 min and allowed to cool for 5 to 10 min. This was repeated three times for a total of 2 h in the boiling water bath. The vial was cooled to room temperature, and the liquid was evaporated with a stream of nitrogen at 50°C. Methylene chloride, 1.5 ml, was applied to the solids to help exclude water and was subsequently removed with a stream of nitrogen gas at room temperature. The vial was crimp sealed with a Teflon-coated butyl rubber stopper and incubated at 50°C for 2 h while the headspace was flushed with nitrogen gas to remove any remaining water. The vial was cooled to room temperature while flushing and then pressurized with nitrogen gas to 83 kPa. A gas-tight, glass syringe was used to add 250 μl of methylene chloride and 250 μl of trifluoroacetic anhydride (TFAA). The vial was vortexed briefly and incubated at room temperature. After 4 h, the vial was chilled on ice, and the majority of the liquid was evaporated by flushing the headspace with a slow stream of nitrogen gas, resulting in a dark brown oil. Because the *N*-trifluoroacetyl amino acid methyl esters are volatile, care was taken not to take the liquid to dryness.

### Preparation of thin-layer chromatography (TLC) standards.

Solutions of 50 mg ml^−1^ of l-methionine (Sigma Aldrich; M9625) and *S*-methyl-l-cysteine (Sigma; M6626) were prepared in 1 M HCl and passed through a 0.2-μm filter. Five hundred microliters of each solution (25 mg of each compound) was added to a serum vial, and the liquid was evaporated with a stream of nitrogen gas at 50°C. The amino acids were then derivatized as described above. A glass syringe was used to add 25 to 50 μl of methyl acetate to dilute the resulting oils.

### TLC purification of l-methionine and *S*-methyl-l-cysteine derivatives.

l-Methionine and *S*-methyl-l-cysteine derivatives were purified as previously described (47). A glass syringe was used to add 150 μl of methyl acetate to dilute the dark brown oil in order to decrease viscosity and ensure complete transfer. A syringe was used to spot all of the liquid from the derivatized cell material as a thin band onto a glass-backed silica gel (500-μm layer) preparative TLC plate (Analtech; P02012). Each standard (5 to 10 μl) was spotted on either side of the band, and all spots were incubated at room temperature for several minutes to dry. A developing chamber was equilibrated with 2.5% methyl acetate in methylene chloride for ≥ 4 h prior to the chromatography. The TLC plate was developed for approximately 40 min or until the solvent front had nearly reached the top of the plate. The plate was allowed to dry completely and was then stained with iodine vapor for several seconds. The positions of the l-methionine and *S*-methyl-l-cysteine standards were marked, and the plate was incubated at room temperature for several minutes until the yellow color from the iodine was no longer visible. The silica gel from the bands of cellular material corresponding to the positions of the standards was transferred to glass serum vials. The vials were crimp sealed with a Teflon-coated butyl rubber stopper, and a glass syringe was used to add 5 ml of methyl acetate. After overnight incubation with gentle shaking, a gas-tight glass syringe was used to transfer the liquid to clean serum vials. The samples were concentrated by evaporating the majority of the liquid with a stream of nitrogen gas, leaving behind 25 to 50 μl of a pale yellow liquid.

### Analysis of methionine and *S*-methyl-l-cysteine derivatives via GC-MS.

TLC-purified l-methionine and l-cysteine derivatives were analyzed via GC-MS at the Proteomics and Mass Spectrometry Facility (University of Georgia) using a modified version of the method described by White (47). One-microliter amounts of the purified derivatives were applied to the injection port (heated at 280°C) of the GC (HP-5890; Agilent) with a splitless duration of 2.75 min and an EC-5 column (0.25-mm inside diameter [i.d.] by 30 m by 0.25-μm film thickness; Alltech). The carrier gas was He with a head pressure capped at 83 kPa. The GC oven was programmed to remain at 60°C for 6 min and then rise from 60°C to 280°C at a rate of 25°C min^−1^. The derivatives were detected using a mass spectrometer (HP-5971A; Agilent) with an electron ionization ion source running in scan mode (monitored *m/z* range, 50 to 350) with 12 scans/s and a detector temperature of 280°C. Under these conditions, the l-methionine derivative (M^+^ = 259) had a retention time of 11.4 to 11.6 min and the *S*-methyl-l-cysteine derivative (M^+^ = 245) had a retention time of 10.6 to 10.9 min. The isotopic composition of all derivatives was measured three times to obtain the error in the GC-MS measurements.

### Calculation of isotopomer enrichments.

The relative percent enrichment of each isotopomer was calculated from the relative abundances observed via GC-MS after correction for the natural abundances of ^2^H, ^13^C, ^15^N, ^17^O, ^18^O, ^33^S, ^34^S, and ^36^S (49) as previously described (50). Briefly, the natural abundance of each mass shift was calculated using a custom R script, and this process was repeated for each of the possible isotopomers for the derivatives of both l-methionine and l-cysteine. The resulting values were then applied to the raw data using the “skewed correction method” (50).

### Correction of legacy material in isotopomer enrichments.

In order to accurately calculate the pathway fluxes and the enrichments of atomic pools (see below), the contributions from “legacy material,” i.e., unlabeled material in the chemostat prior to the introduction of DMSP, needed to be excluded. The amount of legacy material in the chemostat at any given time is defined in the equation below:

(1) $$2^{-t/d}$$

where t is the amount of time (in minutes) that has elapsed after the introduction of DMSP and d is the doubling time of the culture (1,040 min). However, the samples were collected over an extended time period, 24 h in most cases, so equation 1 was integrated with respect to t to determine the amount of legacy material collected between time points a and b. Thus, the amount of legacy material present in a sample (L) collected between times a and b was as follows:

(2) $$L=\frac{-\frac{d}{\text{ln}(2)}\left(2^{-b/d}-2^{-a/d}\right)}{b-a}$$

After calculating the isotopomer enrichments (see above), the legacy material would possess an enrichment of 100% for isotopomers with a mass shift of zero and 0% enrichment of isotopomers with mass shifts greater than zero. Because of this, the observed enrichment of isotopomers with a mass shift of zero would be equal to the following equation:

(3) $$E_{0}=L\cdot 1+(1-L)\cdot {E\prime }_{0}$$

where L is the fraction of legacy material in the sample as defined by equation 2, $E_{0}$ is the observed enrichment of isotopomers with a mass shift of zero, and ${E\prime }_{0}$ is the enrichment of isotopomers with a mass shift of zero in the nonlegacy material only. Similarly, the observed enrichments of mass shifts greater than zero would be equal to the following equation:

(4) $$E_{n}=L\cdot 0+(1-L)\cdot {E\prime }_{n}$$

where $E_{n}$ is the observed enrichment of any isotopomer with a mass shift of n that is greater than zero and ${E\prime }_{n}$ is the enrichment of that mass shift in the nonlegacy material only. Solving for equations 3 and 4 produces the following equations for calculating the isotopomer enrichments in the nonlegacy material only:

(5) $${E\prime }_{0}=\frac{E_{0}-L}{1-L}$$

(6) $${E\prime }_{n}=\frac{E_{n}}{1-L}$$

Equations 5 and 6 were used to calculate the enrichments of each isotopomer in the nonlegacy material for each sample.

### Calculation of the specific labeling of the sulfide pool and the l-serine pool.

Because l-cysteine is synthesized exclusively through the random reassembly of sulfide and carbon (Fig. 2), the sulfide pool can be approximated with the following equations:

(7) $$\frac{S_{U}}{S_{L}}=\frac{c_{0}+c_{1}}{c_{2}+c_{3}}$$

(8) $$S_{U}+S_{L}=1$$

where $S_{U}$ is the fraction of unlabeled sulfide, $S_{L}$ is the fraction of labeled sulfide, $c_{0}$ is the enrichment of unlabeled l-cysteine, $c_{1}$ is the enrichment of ^13^C-labeled l-cysteine, $c_{2}$ is the enrichment of ^34^S-labeled l-cysteine, and $c_{3}$ the enrichment of doubly labeled l-cysteine, all in nonlegacy material. After solving equations 7 and 8 for $S_{U}$ and $S_{L}$, the following equations were obtained:

(9) $$S_{L}={\left(\frac{c_{0}+c_{1}}{c_{2}+c_{3}}+1\right)}^{-1}$$

(10) $$S_{U}=1-S_{L}$$

These equations were applied to each replicate prior to calculating the average fraction of labeled sulfide for each sample.

The carbon of l-cysteine is derived from l-serine, so the ^13^C labeling of the l-serine pool can be approximated from the ^13^C labeling of l-cysteine with the following equations:

(11) $$\frac{{\text{Ser}}_{U}}{{\text{Ser}}_{L}}=\frac{c_{0}+c_{2}}{c_{1}+c_{3}}$$

(12) $${\text{Ser}}_{U}+{\text{Ser}}_{L}=1$$

where ${\text{Ser}}_{U}$ is the fraction of unlabeled l-serine, and ${\text{Ser}}_{L}$ is the fraction of labeled l-serine. After solving equations 11 and 12 for ${\text{Ser}}_{U}$ and ${\text{Ser}}_{L}$, the following equations were obtained:

(13) $${\text{Ser}}_{L}={\left(\frac{c_{0}+c_{2}}{c_{1}+c_{3}}+1\right)}^{-1}$$

(14) $${\text{Ser}}_{U}=1-{\text{Ser}}_{L}$$

These equations were applied to each replicate prior to calculating the average fraction of labeled l-serine.

### Calculation of the specific labeling of the methyl-THF pool and the fraction of l-methionine synthesized via the reassembly pathway.

It was assumed that the sulfide pool used for l-cysteine biosynthesis was equivalent to the sulfide pool used for the reassembly pathway of l-methionine biosynthesis. However, l-cysteine is synthesized via l-serine, which is in turn formed from either glucose via 3-phosphoglycerate or glycine and methylene-THF. Thus, the C-1-THF pool cannot be estimated using the observed enrichments for l-cysteine (Fig. 2). Because singly labeled l-methionine can be synthesized only via the reassembly pathway (Fig. 2 and 3), the following equations were assumed to be true:

(15) $$m_{1}=C_{L}S_{U}F_{R}$$

(16) $$m_{2}=C_{U}S_{L}F_{R}$$

(17) $$C_{U}+C_{L}=1$$

where $S_{U}$ and $S_{L}$ are the fractions of unlabeled and labeled sulfide, respectively, as calculated from the l-cysteine labeling in equations 9 and 10, $C_{U}$ is the fraction of unlabeled methyl-THF, $C_{L}$ is the fraction of labeled methyl-THF, $F_{R}$ is the fraction of l-methionine synthesized via the reassembly pathway, $m_{1}$ is the enrichment of ^13^C-labeled l-methionine in nonlegacy material, and $m_{2}$ is the enrichment of ^34^S-labeled l-methionine in nonlegacy material. In order to solve for $C_{U}$ and $C_{L}$, equations 15 and 16 were combined to produce the following equation:

(18) $$\frac{m_{1}}{m_{2}}=\frac{C_{L}S_{U}}{C_{U}S_{L}}$$

To solve for $C_{U}$ and $C_{L}$ in equations 17 and 18, the following equations were obtained:

(19) $$C_{U}=\frac{m_{2}S_{U}}{m_{1}S_{L}+m_{2}S_{U}}$$

(20) $$C_{L}=1-C_{U}$$

Next, $C_{U}$ in equation 16 was replaced with the term defined in equation 19, and the resulting equation was solved for $F_{R}$:

(21) $$F_{R}=\frac{m_{1}S_{L}+m_{2}S_{U}}{S_{U}S_{L}}$$

Equations 19 and 20 were used to calculate the specific labeling of the methyl-THF pool, and equation 21 was used to calculate the fraction of l-methionine synthesized via the reassembly pathway.

### Calculation of the specific labeling of the methanethiol pool and the fraction of l-methionine synthesized via the direct capture pathway.

Because unlabeled l-methionine ($m_{0}$) and doubly labeled l-methionine ($m_{3}$) can be biosynthesized by both the reassembly pathway and the direct capture pathway (Fig. 2 and 3), the following equations were assumed to be true:

(22) $$m_{0}=C_{U}S_{U}F_{R}+M_{U}F_{D}$$

(23) $$m_{3}=C_{L}S_{L}F_{R}+M_{L}F_{D}$$

(24) $$F_{D}+F_{R}=1$$

where $M_{U}$ is the fraction of unlabeled methanethiol, $M_{L}$ is the fraction of doubly labeled methanethiol, $F_{D}$ is the fraction of l-methionine synthesized via the direct capture pathway, $m_{0}$ is the enrichment of unlabeled l-methionine in nonlegacy material, and $m_{3}$ is the enrichment of doubly labeled l-methionine in nonlegacy material. Solving equations 22 to 24 for $M_{U}$, $M_{L}$, and $F_{D}$ produces the following equations:

(25) $$M_{U}=\frac{m_{0}-C_{U}S_{U}F_{R}}{1-F_{R}}$$

(26) $$M_{L}=\frac{m_{3}-C_{L}S_{L}F_{R}}{1-F_{R}}$$

(27) $$F_{D}=1-F_{R}$$

Equations 25 and 26 were used to calculate the specific enrichment of the methanethiol pool, and equation 27 was used to calculate the fraction of l-methionine synthesized via the direct capture pathway.
