# Supplementary material for: Dimethylsulfoniopropionate Sulfur and Methyl Carbon Assimilation in Ruegeria Species
Source: mBio. 2020 Mar 24;11(2):e00329-20. doi: 10.1128/mBio.00329-20 (PMC7157515; doi:10.1128/mBio.00329-20)
Supplement: TABLE S1 [file mBio.00329-20-st001.pdf]

Dimethylsulfoniopropionate sulfur and methyl carbon assimilation in *Ruegeria* species

Joseph S. Wirth, Tao Wang, Qiuyuan Huang, Robert H. White, and William B. Whitman

Supplementary Material

Table S1. DMSP genes in *Ruegeria pomeroyi* DSS-3 and *Ruegeria lacuscaerulensis* ITI-1157[a].

Reaction[b]	Gene	*R. pomeroyi* DSS-3	*R. lacuscaerulensis* ITI-1157
1	*dddD*	SPO1703	—
1	*dddP*	SPO2299	SL1157_2466
1	*dddQ*	SPO1596	SL1157_0332
1	*dddW*	SPO0453	—
2	*prpE*	SPO2934	SL1157_2163
3	*acuI*	SPO1914	SL1157_2966
4	*dmdA*	SPO1913	SL1157_2967
5	*dmdB*	SPO2045, SPO0677	SL1157_1815, SL1157_2728
6	*dmdC*	SPO3804, SPO0298, SPO2915	SL1157_0694, SL1157_2180
7	*dmdD*	SPO3805	—
7	*acuH*	SPO0147	SL1157_0807
8	*aldH*	SPO0097	SL1157_1245
9	*mtoX*	SPOA0269	—

[a]: Locus tags for the pathways detailed in **figure 1** are shown. Multiple locus tags indicate the presence of additional homologues. "—" indicates the absence of a gene homologue.

[b]: Numbers reference the reactions depicted in **figure 1**.
