# Supplementary material for: Dimethylsulfoniopropionate Sulfur and Methyl Carbon Assimilation in Ruegeria Species
Source: mBio. 2020 Mar 24;11(2):e00329-20. doi: 10.1128/mBio.00329-20 (PMC7157515; doi:10.1128/mBio.00329-20)
Supplement: TABLE S2 [file mBio.00329-20-st002.pdf]

Dimethylsulfoniopropionate sulfur and methyl carbon assimilation in *Ruegeria* species

Joseph S. Wirth, Tao Wang, Qiuyuan Huang, Robert H. White, and William B. Whitman

Supplementary Material

Table S2. Comparison of the relative abundances of mass shifts for *R. pomeroyi* and *R. lacuscaerulensis* chemostat cultures.

Compound	Mass Shift	*R. pomeroyi*[a]	*R. lacuscaerulensis*[a]
Methionine	0	0.418 ± 0.002	0.428 ± 0.044
	1	0.075 ± 0.004	0.070 ± 0.010
	2	0.258 ± 0.010	0.232 ± 0.009
	3	0.249 ± 0.007	0.260 ± 0.018
Cysteine	0	0.416 ± 0.025	0.428 ± 0.033
	1	0.099 ± 0.035	0.041 ± 0.025
	2	0.376 ± 0.012	0.478 ± 0.056
	3	0.106 ± 0.021	0.053 ± 0.007

[a]: Values indicate the mean (n = 3) relative abundance of each mass shift after five days in chemostat following the addition of 50 µM DMSP (50.18 ± 2.24 % enriched with [^{13}C][^{34}S]DMSP) to the chemostat reservoir. Error indicates the 95 % confidence interval.
