# Supplementary material for: Dimethylsulfoniopropionate Sulfur and Methyl Carbon Assimilation in Ruegeria Species
Source: mBio. 2020 Mar 24;11(2):e00329-20. doi: 10.1128/mBio.00329-20 (PMC7157515; doi:10.1128/mBio.00329-20)
Supplement: TABLE S3 [file mBio.00329-20-st003.pdf]

Dimethylsulfoniopropionate sulfur and methyl carbon assimilation in *Ruegeria* species

Joseph S. Wirth, Tao Wang, Qiuyuan Huang, Robert H. White, and William B. Whitman

Supplementary Material

Table S3. Comparison of the isotopomer enrichments for *R. pomeroyi* and *R. lacuscaerulensis* chemostat cultures.

Compound	Isotopomer	*R. pomeroyi*[a]	*R. lacuscaerulensis*[a]
Methionine	$^{12}C^{32}S$	47.8 ± 0.2	49.0 ± 5.2
	$^{13}C^{32}S$	3.9 ± 0.5	3.2 ± 1.7
	$^{12}C^{34}S$	25.7 ± 1.1	22.9 ± 0.9
	$^{13}C^{34}S$	22.7 ± 0.8	25.4 ± 4.6
	total ^{13}C	26.5 ± 1.0	28.5 ± 6.4
	total ^{34}S	48.3 ± 0.6	48.3 ± 4.5
Cysteine	$^{12}C^{32}S$	47.0 ± 2.9	48.4 ± 3.8
	$^{13}C^{32}S$	7.1 ± 4.2	0.5 ± 1.7
	$^{12}C^{34}S$	38.4 ± 1.7	49.9 ± 6.5
	$^{13}C^{34}S$	7.8 ± 2.9	1.4 ± 0.8
	total ^{13}C	14.9 ± 4.2	1.9 ± 2.3
	total ^{34}S	46.2 ± 3.6	51.3 ± 6.2

[a]: Values indicate the mean (n = 3) percent enrichment of each isotopomer after five days in chemostat following the addition of 50 µM DMSP (50.18 ± 2.24 % enriched with [^{13}C][^{34}S]DMSP) to the chemostat reservoir and were calculated as described in the Materials and Methods. Error indicates the 95 % confidence intervals.
