# Supplementary material for: Dimethylsulfoniopropionate Sulfur and Methyl Carbon Assimilation in Ruegeria Species
Source: mBio. 2020 Mar 24;11(2):e00329-20. doi: 10.1128/mBio.00329-20 (PMC7157515; doi:10.1128/mBio.00329-20)
Supplement: TABLE S4 [file mBio.00329-20-st004.pdf]

Dimethylsulfoniopropionate sulfur and methyl carbon assimilation in *Ruegeria* species

Joseph S. Wirth, Tao Wang, Qiuyuan Huang, Robert H. White, and William B. Whitman

Supplementary Material

Table S4. Comparison of the enriched pools and pathway fluxes for *R. pomeroyi* and *R. lacuscaerulensis* chemostat cultures.

	R. pomeroyi[a]	*R. lacuscaerulensis*[a]
$^{13}CH_3$-THF	11.4 ± 1.6	12.6 ± 6.3
[^{13}C]serine	14.8 ± 4.2	1.9 ± 2.3
$^{34}S^{2-}$	46.0 ± 3.2	51.2 ± 5.7
[^{13}C][^{34}S]methanethiol	52.3 ± 1.9	45.3 ± 8.7
Reassembly pathway	62.9 ± 1.8	51.4 ± 3.4
Direct capture pathway	37.1 ± 1.8	48.6 ± 3.4

[a]: Values indicate the mean (n = 3) percentage of each pool that was enriched or the percentage of L-methionine synthesized via the respective pathway after five days in chemostat following the addition of 50 µM DMSP (50.18 ± 2.24 % enriched with [^{13}C][^{34}S]DMSP) to the chemostat reservoir. Values were calculated as described in the Materials and Methods. Error indicates the 95 % confidence interval.
